# Supplementary material for: Extraction of Acetogenins Using Thermosonication-Assisted Extraction from Annona muricata Seeds and Their Antifungal Activity
Source: Molecules. 2022 Sep 16;27(18):6045. doi: 10.3390/molecules27186045 (PMC9502848; doi:10.3390/molecules27186045)
Supplement: Supplementary file 1 [file molecules-27-06045-s001.zip › molecules-1887392-supplementary.pdf]

molecules

Supplementary Materials

Extraction of Acetogenins Using Thermosonication-Assisted Extraction from *Annona muricata* Seeds and Their Antifungal Activity

Brandon Alexis López-Romero [1], Gabriel Luna-Bárcenas [2], María de Lourdes García-Magaña [1], Luis Miguel Anaya-Esparza [3], Luis Gerardo Zepeda-Vallejo [4], Ulises Miguel López-García [1], Rosa Isela Ortiz-Basurto [1], Gabriela Aguilar-Hernández [3,*], Alejandro Pérez-Larios [3,*] and Efigenia Montalvo-González [1,*]

[1] Laboratorio Integral de Investigación en Alimentos, Tecnológico Nacional de México/Instituto Tecnológico de Tepic, Av. Tecnológico 2595, Lagos del Country, Tepic 63175, Nayarit, Mexico
[2] Centro de Investigación y de Estudios Avanzados, Libramiento Norponiente 2000, Fracc. Real de Juriquilla, Santiago de Querétaro, Querétaro 76230, Mexico
[3] División de Ciencias Agropecuarias e Ingenierías, Centro Universitario de los Altos, Universidad de Guadalajara, Av. Rafael Casillas Aceves 1200, Guadalajara 47600, Jalisco, Mexico
[4] Departamento de Química Orgánica, Escuela Nacional de Ciencias Biológicas, Instituto Politecnico Nacional, Av. Prolongación de Carpio y Plan de Ayala s/n, Col. Santo Tomás, Delegación Miguel Hidalgo, Ciudad de Mexico 11340, Mexico
* Correspondence: gaby.mca2017@gmail.com (G.A.-H.); alarios@cualtos.udg.mx (A.P.-L.); emontalvo@ittepic.edu.mx (E.M.-G.); Tel.: +52-311-211-9400 (E.M.-G.)

Table S1. Temperature profile of the extraction medium using Box-Behnken design.

Time (min)	Temperature (60°C)
0	59 ± 1
10	59 ± 2
20	58 ± 2
30	59 ± 2
40	60 ± 2
50	59 ± 2
Time (min)	Temperature (50°C)
0	50 ± 1
10	49 ± 2
20	49 ± 2
30	48 ± 2
40	50 ± 2
50	50 ± 2
Time (min)	Temperature (40°C)
0	40 ± 1
10	40 ± 2
20	40 ± 2
30	39 ± 2
40	38 ± 2
50	40 ± 2

Temperatures were controlled with a cold-water recirculating bath.
